# Coronary CTA-based radiomic signature of pericoronary adipose tissue predict rapid plaque progression

**DOI:** 10.1186/s13244-024-01731-7

**Published:** 2024-06-20

**Authors:** Yue Li, Huaibi Huo, Hui Liu, Yue Zheng, Zhaoxin Tian, Xue Jiang, Shiqi Jin, Yang Hou, Qi Yang, Fei Teng, Ting Liu

**Affiliations:** 1 Department of Radiology, The First Hospital of China Medical University, Shenyang, China; 2 Department of Radiology, Shengjing Hospital of China Medical University, Shenyang, China; 3 Department of Radiology, Beijing Chaoyang Hospital, Capital Medical University, Beijing, China; 4 Department of Radiology, Chinese Academy of Medical Sciences Fuwai Hospital Shenzhen Hospital, Shenzhen, China

**Keywords:** Radiomic analysis, Rapid plaque progression, Coronary computed tomography angiography, Pericoronary adipose tissue

## Abstract

**Objectives:**

To explore the value of radiomic features derived from pericoronary adipose tissue (PCAT) obtained by coronary computed tomography angiography for prediction of coronary rapid plaque progression (RPP).

**Methods:**

A total of 1233 patients from two centers were included in this multicenter retrospective study. The participants were divided into training, internal validation, and external validation cohorts. Conventional plaque characteristics and radiomic features of PCAT were extracted and analyzed. Random Forest was used to construct five models. Model 1: clinical model. Model 2: plaque characteristics model. Model 3: PCAT radiomics model. Model 4: clinical + radiomics model. Model 5: plaque characteristics + radiomics model. The evaluation of the models encompassed identification accuracy, calibration precision, and clinical applicability. Delong’ test was employed to compare the area under the curve (AUC) of different models.

**Results:**

Seven radiomic features, including two shape features, three first-order features, and two textural features, were selected to build the PCAT radiomics model. In contrast to the clinical model and plaque characteristics model, the PCAT radiomics model (AUC 0.85 for training, 0.84 for internal validation, and 0.81 for external validation; *p* < 0.05) achieved significantly higher diagnostic performance in predicting RPP. The separate combination of radiomics with clinical and plaque characteristics model did not further improve diagnostic efficacy statistically (*p* > 0.05).

**Conclusion:**

Radiomic feature analysis derived from PCAT significantly improves the prediction of RPP as compared to clinical and plaque characteristics. Radiomic analysis of PCAT may improve monitoring RPP over time.

**Critical relevance statement:**

Our findings demonstrate PCAT radiomics model exhibited good performance in the prediction of RPP, with potential clinical value.

**Key Points:**

- Rapid plaque progression may be predictable with radiomics from pericoronary adipose tissue.
- Fibrous plaque volume, diameter stenosis, and fat attenuation index were identified as risk factors for predicting rapid plaque progression.
- Radiomics features of pericoronary adipose tissue can improve the predictive ability of rapid plaque progression.

**Graphical Abstract:**

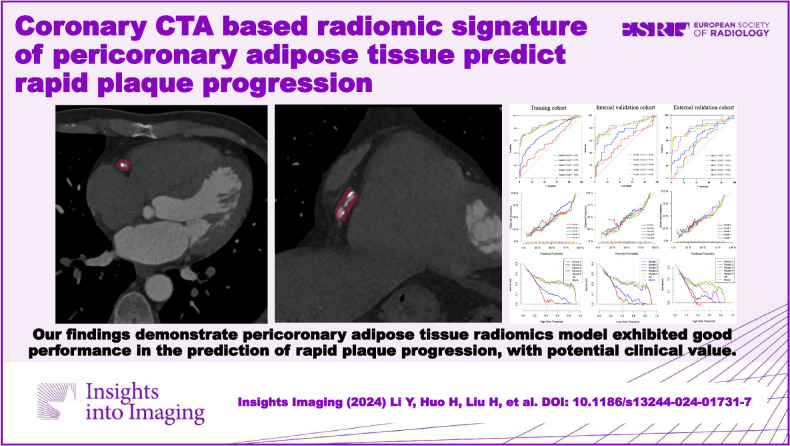

## Introduction

The consequences of coronary artery disease are the leading cause of mortality worldwide [1]. Atherosclerotic plaque formation and rapid plaque progression (RPP) are the main underlying drivers in coronary artery disease [2–4]. Studies have shown that coronary plaque tends to increase rapidly in the months prior to an acute coronary event, and this phenomenon of plaque progression is the prerequisite for plaque rupture [5].

Wall inflammation is a major contributor to atherosclerotic plaque instability, which can promote the progression and rupture of coronary plaques [6–8]. A persistent two-way interaction between the coronary wall and the surrounding pericoronary adipose tissue (PCAT) has been demonstrated. The fat attenuation index (FAI) of pericoronary fat has been used as an indirect marker reflecting coronary inflammation by estimating the mean density value of pericoronary fat at coronary computed tomography angiography (CCTA) [7, 9–11]. However, fat density primarily relies on the values of voxel intensity, whereas radiomic analysis may provide a more detailed analysis of voxel characteristics [12].

Radiomic analysis extracts a large number of quantitative features (such as shape, attenuation, intensity distribution, and spatial information) from medical images, which can be used to quantitatively evaluate the heterogeneity of lesions, thereby improving diagnostic accuracy [13–15]. Recently, the radiomic analysis of PCAT has been shown to improve the prediction of acute coronary syndromes [16, 17]. However, whether the radiomic analysis of PCAT can improve the accuracy of RPP prediction is largely unknown.

Accordingly, the purpose of this study is to explore the value of radiomic analysis of PCAT in the prediction of RPP.

## Materials and methods

This retrospective study was approved by the ethics committee (IRB number: KT2021213), and the requirement for written informed consent was waived.

### Study population

**Fig. 1:**
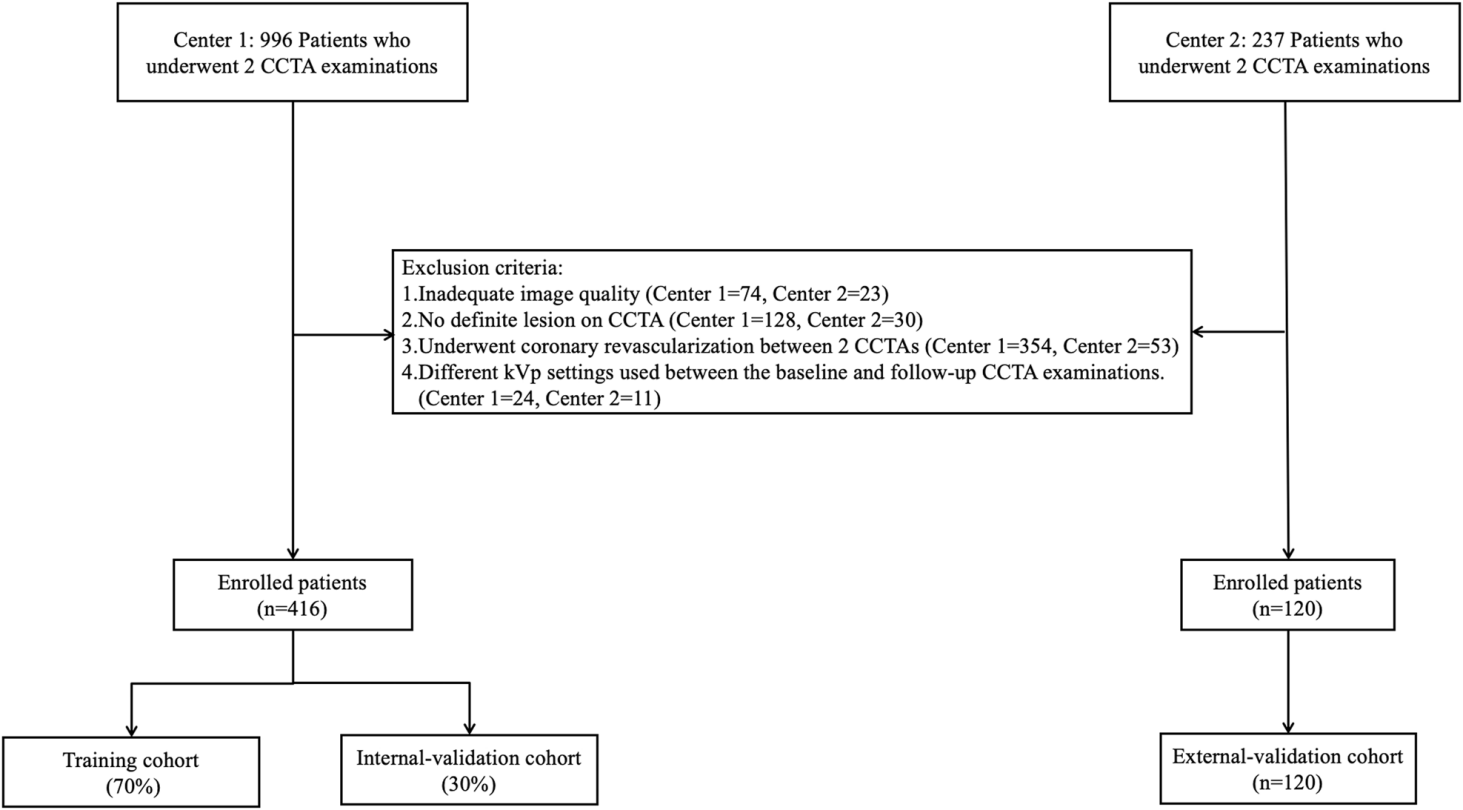
A flowchart of patient recruitment and study design. CCTA, coronary computed tomography angiography

Patients from two centers who underwent two CCTA examinations were enrolled in this retrospective study. All included patients in Center 1 from January 2016 to August 2022 were split into training and internal validation cohorts at random in a 7:3 ratio. Patients in Center 2 from January 2018 and December 2022 were assigned to an external validation cohort. Inclusion criteria were: (1) patients with known or suspected coronary artery disease; (2) patients undergoing two CCTA scans performed with the same CT equipment; (3) the interval between two CCTA examinations was longer than 6 months. The exclusion criteria of patients were: (1) inadequate image quality for plaque analysis; (2) lack of visible lesions on CCTA; (3) patients who underwent coronary artery bypass grafting or coronary stent implantation between two CCTAs; (4) different kVp settings used between the baseline and follow-up CCTA examinations. The flowchart of patient selection is shown in Fig. 1.

### CCTA acquisition

All scans of the two centers were conducted using a second-generation dual-source CT unit (Somaton Definition Flash CT, Siemens Healthcare). The target heart rate was 60–80 beats/min, and patients with a heart rate > 80 beats/min were given an oral *β*-blocker 1 h before the examination. Sublingual nitroglycerin was administered to each patient within 3–5 min before the start of the scan. Retrospective ECG-triggered was used for coronary image acquisition. 50–100 mL of nonionic iodine contrast agent was injected into the antecubital vein at a rate of 4–5 mL/s using a dual-channel high-pressure syringe. Then followed by a 50 mL saline flush. The CCTA acquisition initiated with a 4-s delay following the ascending aorta’s peak time. Parameters for acquisition and reconstruction of the two centers included: a tube voltage of 120 kVp, tube current automatically adjusted according to patient BMI, 60 × 0.6 mm collimation, and 0.75 mm reconstructed slice thickness.

### CCTA analysis

Analysis of coronary segments was conducted on vessels ≥ 2 mm in diameter based on the 17-segment model [18]. Analysis of baseline and follow-up coronary plaques were performed at the highest-grade stenosis using semi-automated software (QAngioCT Research Edition v3.2.0.13; Medis Medical Imaging Systems). The software automatically recognizes the contours of the lumen and vessel, with manual adjustment as needed. Fiduciary landmarks were used to coregister baseline and follow-up coronary segments, such as distance from branch vessel origins or ostia.

Qualitative plaque features were analyzed, including positive remodeling, spotty calcifications, low-attenuation plaques, and the napkin-ring sign [19, 20]. High-risk plaque was defined as lesions with ≥ 2 features above.

Quantitative plaque characteristics included diameter stenosis and total plaque volume. Plaque characteristics were subclassified as necrosis core (−30–30 Hounsfield units (HU)), fibrofatty plaque (31–130 HU), fibrous plaque (131–350 HU), and calcified plaque (> 350 HU) [21, 22]. Plaque burden (PB) was defined as plaque volume divided by vessel volume [23]. Voxels within a radial distance equal to the average diameter of the corresponding coronary vessel and showing CT attenuation values ranging from −190 to −30 HU were identified as PCAT, and the software-generated fat measurements FAI (−190–30 HU) [24, 25]. According to the annual change in PB ((follow-up PB − baseline PB)/CCTA intervals * 100% > 1.0%) was defined as RPP [26–29]. All images were assessed by two radiologists separately with 5 years’ of work experience. Each parameter was measured three times, and the average value was used as the final result.

### Radiomic analysis; Image segmentation and radiomic features analysis

For segmentation and radiomic feature analysis, all images were transferred into the Research Portal V1.1 (United Imaging Intelligence, Co., Ltd.). The region of interest (ROI) was outlined manually layer by layer for pericoronary fat adjacent to the plaque lesion separately. For each lesion, 1904 radiomics parameters were extracted in total, including first-order features, shape features, gray-level co-occurrence matrix (GLCM), gray-level run-length matrix (GLRLM), gray-level size-zone matrix (GLSZM), gray-level dependence matrix (GLDM), and neighboring gray-tone difference matrix (NGTDM). In the process of feature extraction, we selected features with an intra-class correlation coefficient value > 0.75 for subsequent analysis, and the feature pairs exhibiting a Spearman’s correlation coefficient above 0.9 were eliminated. Finally, the least absolute shrinkage and selection operator (LASSO) method was used for refining feature analysis.

### Model construction and validation

**Fig. 2:**
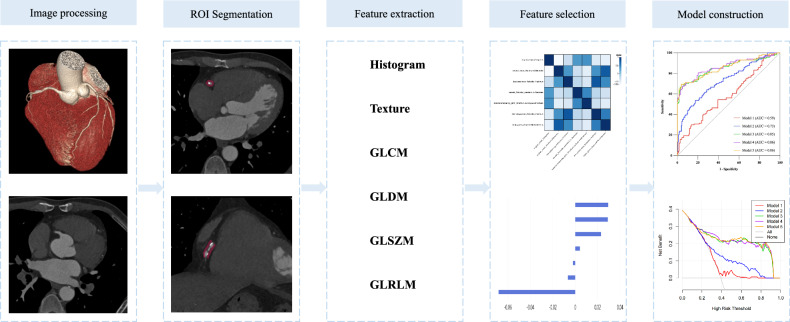
A flowchart of the prediction model development process

We developed five models to predict RPP. Model 1: clinical model. Model 2: plaque characteristics model. Model 3: PCAT radiomics model. Model 4: clinical + radiomics model. Model 5: plaque characteristics + radiomics model. Random Forest was used to build models. To assess the performance of the models, decision curve analysis (DCA), calibration curve analysis, and receiver operating characteristic (ROC) analyses were conducted. The workflow of the radiomics is displayed in Fig. 2.

### Statistical analysis

All statistical analyses were conducted utilizing SPSS software (version 26.0, IBM) and the R software (version 4.1.2). Continuous variables were expressed as means ± standard deviations (SD) or median (interquartile range). To assess the variances in continuous variables between the two groups, the student’s *t*-test and Mann–Whitney *U*-test were employed. Frequencies and percentages were used to represent categorical variables, and the Chi-square test was utilized to compare the two groups. Univariate and multivariate logistic analyses were employed to identify factors influencing the RPP. The effectiveness of different models was assessed using the area under the curve (AUC) of the ROC analysis. The AUC of different models was compared using the Delong test. Statistical significance was indicated by a two-sided *p*-value < 0.05.

## Results

### Baseline clinical characteristics

Table 1 summarized the clinical characteristics of the three cohorts. A comparison of clinical characteristics between the RPP group and non-RPP group was presented in Table 2. There were no significant differences in the clinical characteristics between the groups with and without RPP in the training and internal validation cohort. In the external validation cohort, the prevalence of smoking was higher in the RPP group than in the non-RPP group.

**Table 1:** Clinical characteristics of three cohorts

Characteristics	Training cohort (*n* = 291)	Interval validation cohort (*n* = 125)	External validation cohort (*n* = 120)
Age (years)	58.3 ± 9.2	58.6 ± 9.4	59.6 ± 7.4
Male, *n* (%)	190 (65.3)	77 (61.6)	64 (53.3)
Intervals time (years)	1.7 (1.1–2.1)	1.7 (1.1–2.3)	1.6 (1.1–2.2)
Smoking, *n* (%)	79 (27.1)	21 (16.8)	42 (35.0)
Hypertension, *n* (%)	150 (51.5)	60 (48.0)	75 (62.5)
Diabetes mellitus, *n* (%)	54 (18.6)	19 (15.2)	32 (26.7)
Family history, *n* (%)	31 (10.7)	8 (6.4)	11 (9.2)
Dyslipidemia, *n* (%)	146 (50.2)	57 (45.6)	63 (52.5)
Symptoms, *n* (%)			
Typical	62 (21.3)	27 (21.6)	32 (26.7)
Atypical	15 (5.2)	5 (4.0)	8 (6.7)
Nonanginal	33 (11.3)	22 (17.6)	15 (12.5)
Other	23 (7.9)	9 (7.2)	16 (13.3)
No symptoms	158 (54.3)	62 (49.6)	49 (40.8)
Medication, *n* (%)			
Aspirin	140 (48.1)	60 (48.0)	45 (37.5)
Statin	167 (57.4)	78 (62.4)	83 (69.2)
Beta-blockers	59 (20.3)	23 (18.4)	29 (24.2)
Lipid profile, mg/dL			
Total cholesterol	4.6 (3.9–5.2)	4.6 (3.9–5.2)	3.9 (3.0–5.0)
Triglycerides	1.3 (1.0–2.0)	1.3 (1.1–1.9)	1.6 (1.3–2.0)
HDL cholesterol	1.2 (1.0–1.4)	1.2 (1.0–1.3)	1.4 (1.2–1.6)
LDL cholesterol	2.9 (2.3–3.4)	2.9 (2.4–3.3)	2.3 (1.7–3.0)

Values are mean ± SD, *n* (%), or median (IQR)

*HDL* high-density lipoprotein, *LDL* low-density lipoprotein

**Table 2:** Comparison of clinical characteristics between the RPP group and non-RPP group of three cohorts

Characteristics	Training cohort (*n* = 291)			Internal validation cohort (*n* = 125)			External validation cohort (*n* = 120)		
	RPP (*n* = 114)	Non-RPP (*n* = 177)	*p*	RPP (*n* = 49)	Non-RPP (*n* = 76)	*p*	RPP (*n* = 39)	Non-RPP (*n* = 81)	*p*
Age (years)	59.33 ± 9.8	57.6 ± 8.8	0.117	58.2 ± 9.7	58.9 ± 9.4	0.703	60.1 ± 6.9	59.4 ± 7.7	0.616
Male, *n* (%)	81 (71.1)	109 (61.6)	0.098	30 (61.2)	47 (61.8)	0.945	24 (61.5)	40 (49.4)	0.211
Intervals time (years)	1.6 (1.1–2.1)	1.7 (1.1–2.2)	0.241	1.4 (1.0–1.9)	1.8 (1.1–2.3)	0.056	1.6 (1.1–2.2)	1.6 (1.1–2.2)	0.582
Smoking, *n* (%)	38 (33.3)	41 (23.2)	0.057	11 (22.4)	10 (13.2)	0.175	19 (48.7)	23 (28.4)	0.029
Hypertension, *n* (%)	63 (55.3)	87 (49.2)	0.309	26 (53.1)	34 (44.7)	0.363	22 (56.4)	53 (65.4)	0.339
Diabetes mellitus, *n* (%)	26 (22.8)	28 (15.8)	0.134	11 (22.4)	8 (10.5)	0.070	14 (35.9)	18 (22.2)	0.113
Family history, *n* (%)	16 (14.0)	15 (8.5)	0.133	5 (10.2)	3 (3.9)	0.261	4 (10.3)	7 (8.6)	0.747
Dyslipidemia, *n* (%)	59 (51.8)	87 (49.2)	0.665	24 (49.0)	33 (43.4)	0.542	23 (59.0)	40 (49.4)	0.324
Symptoms, *n* (%)									
Typical	21 (18.4)	41 (23.2)	0.335	9 (18.4)	18 (23.7)	0.481	9 (23.1)	23 (28.4)	0.537
Atypical	7 (6.1)	8 (4.5)	0.542	3 (6.1)	2 (2.6)	0.379	4 (10.3)	4 (4.9)	0.274
Nonanginal	14 (12.3)	19 (10.7)	0.685	11 (22.4)	11 (14.5)	0.253	7 (17.9)	8 (9.9)	0.244
Other	6 (5.3)	17 (9.6)	0.180	2 (4.1)	7 (9.2)	0.481	5 (12.8)	11 (13.6)	0.909
No symptoms	66 (57.9)	92 (52.0)	0.323	24 (49.0)	38 (50.0)	0.911	14 (35.9)	35 (43.2)	0.445
Medication, *n*(%)									
Aspirin	58 (50.9)	82 (46.3)	0.448	20 (40.8)	40 (52.6)	0.193	16 (41.0)	29 (35.8)	0.580
Statin	67 (58.8)	100 (56.5)	0.702	29 (59.2)	49 (64.5)	0.551	29 (74.4)	54 (66.7)	0.393
Beta-blockers	19 (16.7)	40 (22.6)	0.219	7 (14.3)	16 (21.1)	0.340	10 (25.6)	19 (23.5)	0.793
Lipid profile, mg/dL									
Total cholesterol	4.6 (3.9–5.2)	4.6 (3.9–5.2)	0.426	4.6 (3.8–5.1)	4.6 (4.0–5.2)	0.867	4.0 (2.9–5.0)	3.8 (3.1–5.1)	0.622
Triglycerides	1.3 (1.0–1.8)	1.3 (1.0–2.0)	0.192	1.4 (1.1–1.9)	1.3 (1.0–1.8)	0.345	1.6 (1.1–2.0)	1.6 (1.4–2.1)	0.310
HDL cholesterol	1.2 (0.9–1.3)	1.2 (1.0–1.4)	0.209	1.2 (0.9–1.3)	1.2 (1.0–1.3)	0.974	1.4 (1.2–1.6)	1.4 (1.2–1.6)	0.906
LDL cholesterol	2.9 (2.1–3.3)	2.9 (2.4–3.5)	0.125	2.9 (2.1–3.2)	2.9 (2.4–3.3)	0.262	2.2 (1.7–3.2)	2.4 (1.7–3.0)	0.701

Values are mean ± SD, *n* (%), or median (IQR)

*RPP* rapid plaque progression, *HDL* high-density lipoprotein, *LDL* low-density lipoprotein

### Conventional coronary plaque characteristics

The conventional coronary plaque characteristics of the three cohorts are presented in Table 3. There were no significant differences in the qualitative plaque characteristics between the RPP group and the non-RPP group. Patients with RPP had significantly higher levels of all the measured quantitative parameters related to plaque, including diameter stenosis, FAI, total plaque volume, fibrous plaque volume, fibrofatty plaque volume, necrotic core volume, and calcified plaque volume than patients without RPP in the training cohort (all *p* < 0.05). There was no statistical difference in calcified plaque volume between the RPP and non-RPP groups in the internal validation cohort. Except for diameter stenosis, fibrous plaque volume, and calcified plaque volume, other quantitative parameters were higher in the RPP group than in the non-RPP group in the external validation cohort. Factors associated with RPP identified in the univariate analysis were presented in Table 4. The multivariate analysis indicated that fibrous plaque volume, diameter stenosis, and FAI were independently associated with RPP.

**Table 3:** Comparison of conventional characteristics between the RPP group and non-RPP group of three cohorts

Characteristics	Training cohort (*n* = 291)			Internal validation cohort (*n* = 125)			External validation cohort (*n* = 120)		
	RPP (*n* = 114)	Non-RPP (*n* = 177)	*p*	RPP (*n* = 49)	Non-RPP (*n* = 76)	*p*	RPP (*n* = 39)	Non-RPP (*n* = 81)	*p*
Positive remodeling, *n* (%)	24 (21.1)	28 (15.8)	0.255	13 (26.5)	10 (13.2)	0.060	13 (33.3)	21 (25.9)	0.399
Spotty calcification, *n* (%)	11 (9.6)	18 (10.2)	0.885	6 (12.2)	8 (10.5)	0.766	10 (25.6)	17 (21.0)	0.567
Napkin-ring sign, *n* (%)	3 (2.6)	2 (1.1)	0.336	2 (4.1)	1 (1.3)	0.561	4 (10.3)	7 (8.6)	0.774
Low-attenuation plaque, *n* (%)	9 (7.9)	15 (8.5)	0.861	6 (12.2)	4 (5.3)	0.188	5 (12.8)	10 (12.3)	0.941
HRP, *n* (%)	18 (15.8)	25 (14.1)	0.696	10 (20.4)	7 (9.2)	0.075	11 (28.2)	16 (19.8)	0.299
DS (%)	40.0 (30.0–56.0)	34.0 (22.0–44.0)	< 0.001	42.0 (30.5–56.0)	36.5 (25.5–49.0)	0.046	44.0 (33.0–57.0)	40.0 (35.0–50.0)	0.209
FAI (HU)	−66.7 ± 7.4	−69.7 ± 8.0	0.002	−65.9 ± 6.7	−69.3 ± 7.3	0.010	−66.3 ± 7.1	−69.2 ± 6.4	0.028
Total plaque volume (mm³)	170.2 (121.1–279.9)	108.5 (48.7–178.6)	< 0.001	205.56 (130.4–280.3)	112.6 (47.5–181.8)	< 0.001	212.3 (134.0–263.7)	151.8 (108.7–208.7)	0.012
Fibrous plaque volume (mm³)	44.4 (20.4–78.9)	27.9 (14.1–42.2)	< 0.001	47.9 (24.0–77.4)	27.6 (14.2–53.4)	0.002	56.0 (23.3–80.9)	39.9 (28.6–57.8)	0.299
Fibrofatty plaque volume (mm³)	24.1 (14.9–48.9)	15.3 (4.8–30.5)	< 0.001	22.6 (12.6–54.9)	11.5 (4.8–26.2)	< 0.001	25.1 (18.0–42.7)	20.2 (5.6–33.9)	0.029
Necrotic core volume (mm³)	64.8 (15.5–101.3)	32.1 (2.7–72.2)	0.001	72.9 (31.7–125.4)	35.5 (2.4–80.6)	0.002	75.6 (39.1–101.6)	43.6 (20.4–85.7)	0.021
Calcified plaque volume (mm³)	28.4 (4.5–56.2)	10.0 (0.3–32.2)	0.001	30.8 (6.1–54.3)	14.0 (1.5–38.7)	0.060	30.0 (0–54.9)	30.0 (8.3–49.6)	0.758
Plaque burden (%)	59.2 (51.0–62.1)	53.6 (41.3–60.0)	< 0.001	59.2 (50.2–64.3)	54.4 (44.1–60.1)	0.035	59.2 (48.8–61.6)	52.5 (44.7–59.2)	0.027
∆PB/y (%/y)	2.9 (1.7–5.2)	−0.3 (–1.3 to 0.1)	< 0.001	2.9 (1.8–6.0)	−0.4 (−1.2 to 0.1)	< 0.001	2.6 (1.8–5.1)	−0.4 (−1.3 to 0.1)	< 0.001

Values are mean ± SD, *n* (%), or median (IQR)

*RPP* rapid plaque progression, *HRP* high-risk plaque, *DS* diameter stenosis, *FAI* fat attenuation index, *PB* plaque burden

**Table 4:** Univariate and multivariate logistic analyses of CCTA-derived parameters predicting rapid plaque progression

Variables	Univariable			Multivariable		
	OR	95% CI	*p*	OR	95% CI	*p*
DS (%)	1.03	1.02–1.05	< 0.001	1.02	1.00–1.04	0.012
FAI (HU)	1.05	1.02–1.08	0.002	1.05	1.01–1.08	0.006
Total plaque volume (mm³)	1.00	1.00–1.01	< 0.001			
Fibrous plaque volume (mm³)	1.02	1.01–1.03	< 0.001	1.02	1.01–1.03	< 0.001
Fibrofatty plaque volume (mm³)	1.02	1.01–1.03	0.002			
Calcified plaque volume (mm³)	1.01	1.01–1.02	< 0.001			
Plaque burden (%)	1.04	1.02–1.06	< 0.001			

*CCTA* coronary computed tomography angiography, *CI* confidence interval, *OR* odds ratio, *DS* diameter stenosis, *FAI* fat attenuation index

### Radiomics feature selection

**Fig. 3:**
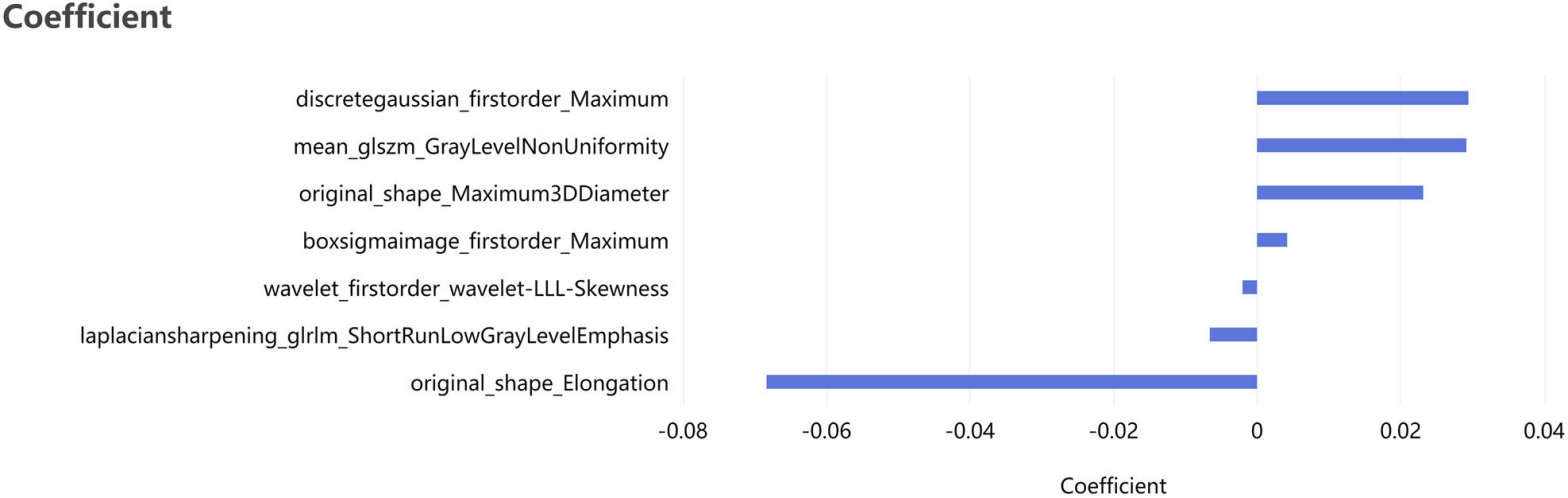
Radiomics feature importance ranking of the seven features most relevant to rapid plaque progression

Among the features extracted in PCAT, seven radiomic features were identified as most valuable in correlation with RPP, notably including original_shape_Elongation, original_shape_Maximum3DDiameter, boxsigmaimage_firstorder_Maximum, wavelet_firstorder_wavelet-LLL-Skewness, laplaciansharpening_glrlm_ShortRunLowGrayLevelEmphasis, discretegaussian_firstorder_Maximum, mean_glszm_GrayLevelNonUniformity. Figure 3 displays the rating of features.

### Predictive performance of models for RPP

#### Discrimination

**Fig. 4:**
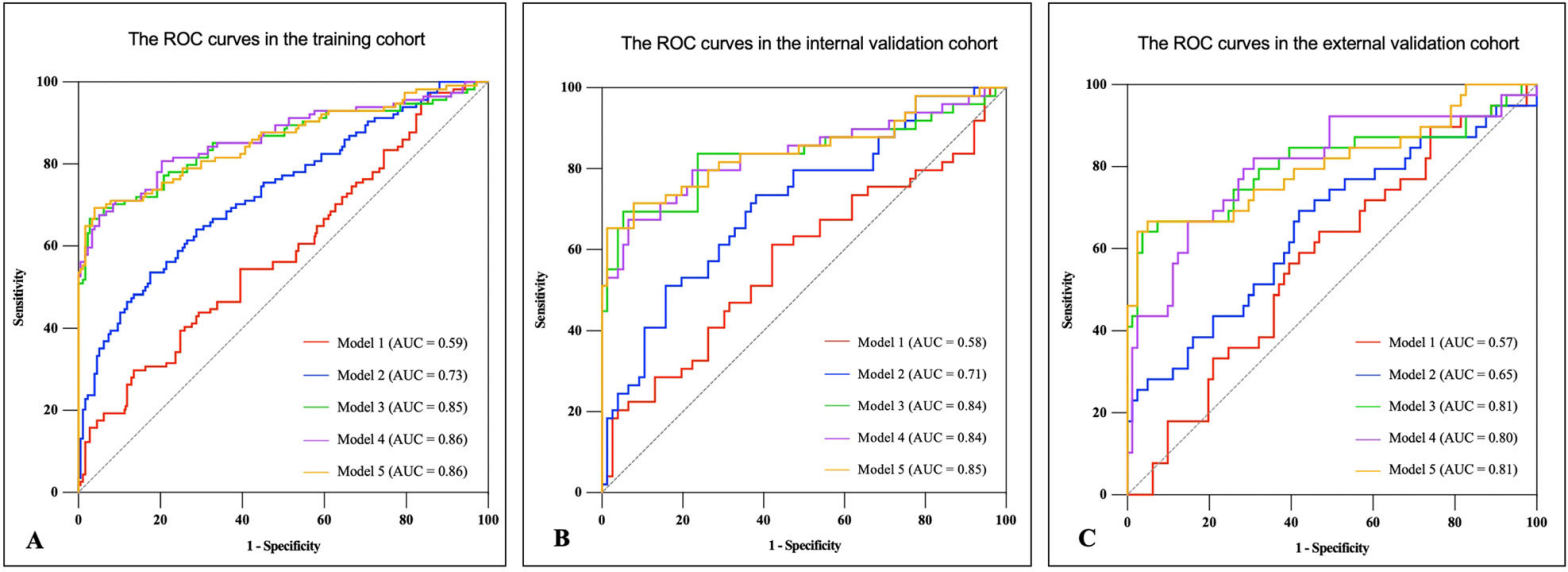
ROC analyses for predicting rapid plaque progression of all models. Model 3, Model 4, and Model 5 exhibited higher AUCs compared to Model 1 and Model 2 in the training (**A**), internal validation (**B**), and external validation (**C**) cohorts. Model 1 = clinical model, Model 2 = plaque characteristics model, Model 3 = PCAT radiomics model, Model 4 = clinical + radiomics model, Model 5 = plaque characteristics + radiomics model. ROC, receiver operating characteristic; AUC, area under the curve; PCAT, pericoronary adipose tissue

For training, internal, and external validation datasets, ROC curves of the five models were constructed to evaluate their efficacy in identifying RPP, as illustrated in Fig. 4. The performance of five predictive models was quantified using metrics such as AUC, sensitivity, specificity, and diagnostic accuracy, detailed in Table 5. We found that the radiomics model performed better than the clinical model and plaque characteristics model in the training, internal validation cohorts, and external validation cohorts. According to DeLong’s test, in the training, internal, and external validation dataset, the AUC of the radiomics model differed significantly from the clinical model and the plaque characteristics model (*p* < 0.05). In the training dataset, Model 4 exhibited a slightly higher AUC compared to Model 3. Similarly, Model 5 demonstrated a marginally higher AUC than Model 3 in both the training and the internal validation datasets. However, according to the DeLong’ test, these differences were not statistically significant.

**Table 5:** Recognition ability of all models for patients with rapid plaque progression

Cohort	Model	AUC (95% CI)	SEN	SPE	ACC
Training	Model 1	0.59 (0.52–0.65)	0.30	0.86	0.64
	Model 2	0.73 (0.67–0.79)	0.64	0.71	0.68
	Model 3	0.85 (0.80–0.90)	0.66	0.97	0.85
	Model 4	0.86 (0.82–0.91)	0.67	0.95	0.84
	Model 5	0.86 (0.81–0.91)	0.69	0.96	0.86
Internal validation	Model 1	0.58 (0.47–0.68)	0.29	0.86	0.63
	Model 2	0.71 (0.62–0.81)	0.65	0.65	0.65
	Model 3	0.84 (0.75–0.92)	0.63	0.96	0.83
	Model 4	0.84 (0.76–0.92)	0.65	0.93	0.82
	Model 5	0.85 (0.77–0.92)	0.67	0.92	0.82
External validation	Model 1	0.57 (0.47–0.68)	0.33	0.78	0.63
	Model 2	0.65 (0.54–0.76)	0.72	0.54	0.60
	Model 3	0.81 (0.71–0.91)	0.62	0.96	0.85
	Model 4	0.80 (0.71–0.90)	0.64	0.85	0.78
	Model 5	0.81 (0.71–0.90)	0.67	0.94	0.85

*Model 1*  clinical model, *Model 2*  plaque characteristics model, *Model 3* PCAT radiomics model, *Model 4* clinical + radiomics model, *Model 5* plaque characteristics + radiomics model, *AUC* area under the curve, *95% CI* 95% confidence interval, *SEN* sensitivity, *SPE* specificity, *ACC* accuracy, *PCAT* pericoronary adipose tissue

#### Calibration

**Fig. 5:**
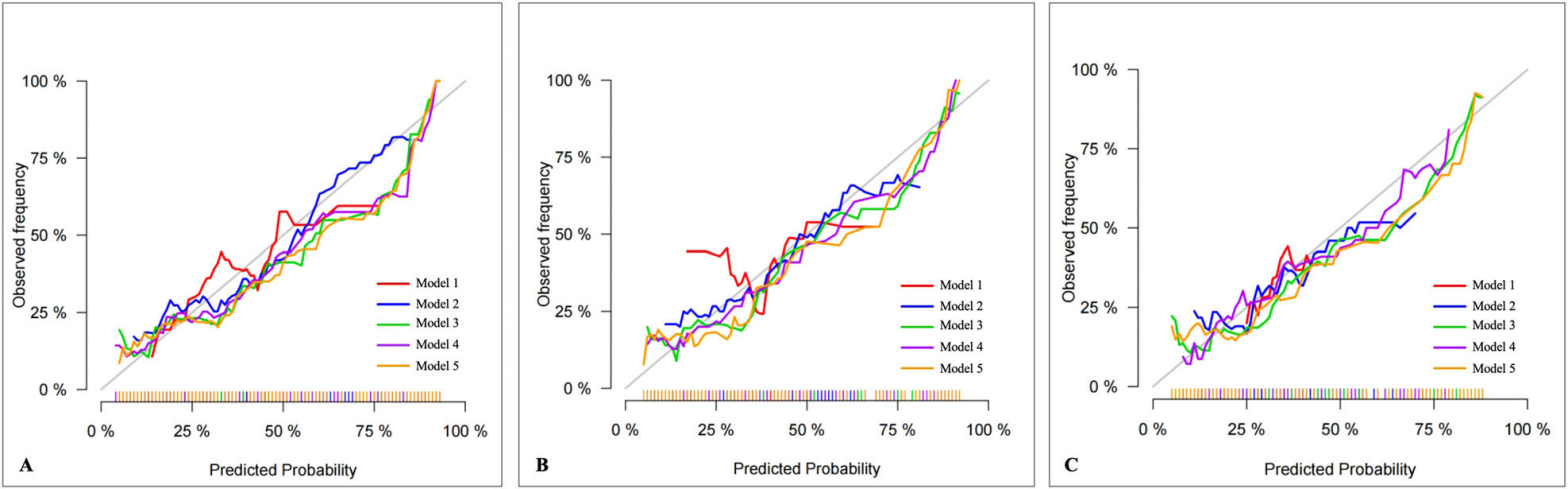
Calibration curves for the five models. All models demonstrated good calibration for predicting rapid plaque progression in the training (**A**), internal validation (**B**), and external validation (**C**) cohorts. Model 1, clinical model; Model 2, plaque characteristics model; Model 3, PCAT radiomics model; Model 4, clinical + radiomics model; Model 5, plaque characteristics + radiomics model, PCAT, pericoronary adipose tissue

The five predictive models’ calibration curves indicated a high degree of alignment between the prediction outcomes and true results in the training, internal, and external validation datasets (Fig. 5).

#### Clinical application

**Fig. 6:**
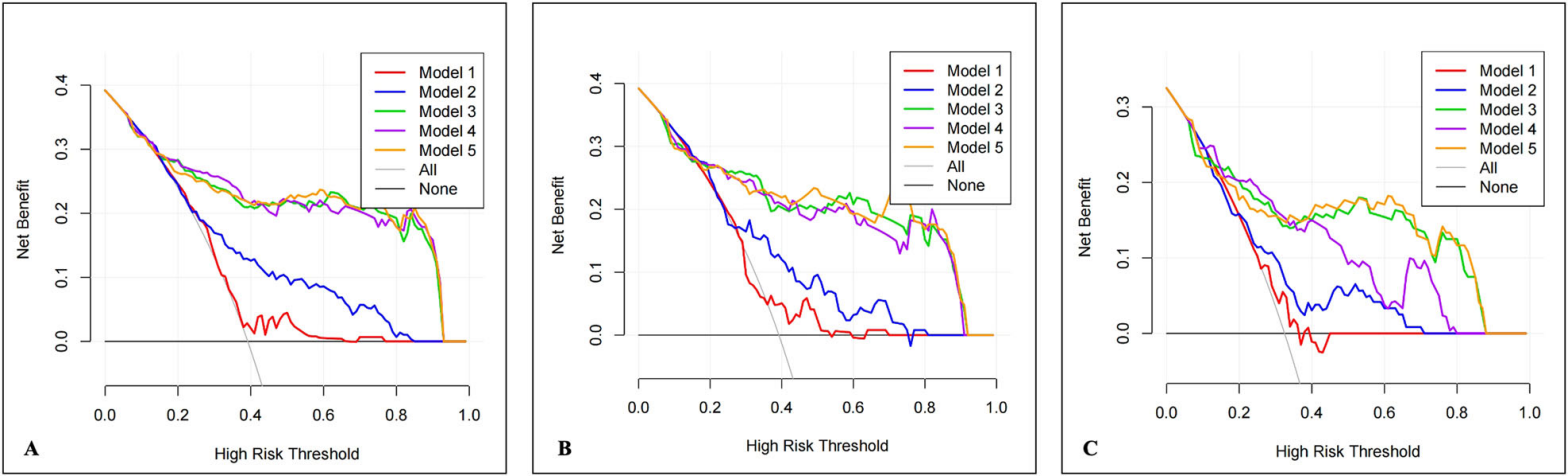
The decision curve analysis for the five models in predicting rapid plaque progression. The Model 3, Model 4, and Model 5 had a higher net benefit than the Model 1 and Model 2 in the training (**A**), internal validation (**B**), and external validation (**C**) datasets. Model 1, clinical model; Model 2, plaque characteristics model; Model 3, PCAT radiomics model; Model 4, clinical + radiomics model; Model 5, plaque characteristics + radiomics model; PCAT, pericoronary adipose tissue

The DCA was employed to ascertain the clinical applicability of the five predictive models by comparing their net benefits at various threshold probabilities within training, internal and external validation datasets, which revealed a superior net benefit for the Model 3, Model 4, and Model 5 over the Model 1 and Model 2 (Fig. 6).

## Discussion

A PCAT radiomics model to predict RPP was tested in the current study. The main result showed that the PCAT radiomics model achieves better performance in predicting RPP as compared with the clinical model and plaque characteristics model. Furthermore, combining plaque characteristics with the radiomics model did not further improve diagnostic efficacy statistically.

In line with the findings of Han et al [30], our study showed statistically significant differences in plaque quantification metrics based on CCTA between groups with and without RPP. When incorporating significant quantitative parameters from univariate analysis into a multivariate analysis, fibrous plaque volume, diameter stenosis, and FAI emerged as independent predictors of RPP. The increase in fibrous plaque volume reflects a cumulative burden of atherosclerosis that contributes to coronary plaque progression. Diameter stenosis can alter blood flow patterns, leading to reduced shear stress in certain areas. In a low-shear stress environment, endothelial cells express more inflammatory factors and adhesion molecules, promoting the adhesion of white blood cells and other inflammatory cells to the vessel wall. This exacerbates the inflammatory response and promotes the progression of plaque [31]. Consistent with our study, a previous study indicated that the increase in vessel inflammation represented by PCAT was independently associated with RPP [32]. These findings suggest that FAI is a more sensitive biomarker capable of dynamically reflecting coronary artery inflammation, underscoring its potential clinical utility in early prediction of plaque progression. Furthermore, in our study, no significant differences were observed in high-risk plaque characteristics, contrasting with previous research that confirmed high-risk plaque features as biomarkers of RPP [27, 33]. This discrepancy could be attributed to the small proportion of patients with high-risk plaque features in our study.

However, the information provided by traditional plaque characteristics on the microenvironment of coronary plaques is limited, which presents challenges in the precise and comprehensive evaluation of RPP. Radiomics provides many high-throughput data, enabling the identification of texture features that reflect voxel spatial relationships and capture the microstructural changes within diseased tissue.

Previous radiomic studies have shown findings consistent with our study. Oikonomou et al [12] identified the radiomics features within PCAT reflecting alterations in adipose tissue, that may be indicative of coronary artery inflammation, fibrosis, and angiogenesis. Additionally, Si et al [16] showed that using PCAT radiomics features based on CCTA for identifying acute myocardial infarction patients. It has been shown that PCAT radiomics has better performance than FAI, for identifying patients with acute myocardial infarction. Consistent with our study, Feng et al [34] revealed that the radiomics signature of plaques offered a more accurate predictive value for plaque progression than traditional parameters.

In our study, LASSO was finally used to select the seven best predictors among the 1904 PCAT radiomic features derived from CCTA, including three first-order features, two shape features, and two textural features. The two first-order features, boxsigmaimage_firstorder_Maximum, and discretegaussian_firstorder_Maximum, reflect the highest signal intensity in the lesion area. The higher value of this feature in the RPP group suggests that there is a region of higher pixel intensities in the PCAT, which may indicate that this region is more biologically active, reflecting an increase in local inflammation that can promote plaque progression. Wavelet_firstorder_wavelet-LLL-Skewness indicating skewness reflects asymmetry in the distribution of pixel intensities. Inflammatory activity or changes in the nature of the adipose tissue may lead to changes in the distribution of pixel intensities. Inflammatory regions show higher pixel intensity values on the image due to increased water content, increased cell density, etc. The negative skewness of this feature in the RPP group indicates that more pixel intensities are concentrated at higher values, possibly reflecting heterogeneity and localized inflammatory activity within the PCAT. The size of the original_shape_Elongation feature, which describes the degree of “elongation” of the shape, provides important information about the morphology and potential stability of the PCAT. This feature was negatively correlated with RPP, with lower values in the RPP group than in the non-RPP group. Smaller values of original_shape_Elongation indicate that the PCAT shape in the RPP group is relatively more irregular, possibly reflecting structural changes in the PCAT that occur during plaque progression, such as an uneven distribution of fat or changes in localized fat volume. This irregularity in shape may result from inflammation, remodeling of adipose tissue, or altered interaction with the vessel wall. Irregularly shaped PCAT may exert uneven external pressure on neighboring coronary arteries, affecting the distribution of stress in the wall and thus affecting plaque progression. The original_shape_Maximum3DDiameter feature measures the distance between the two farthest points within the ROI in three-dimensional space, considering the length, width, and height dimensions, and provides information about the overall size of the lesion area. A larger value of this feature indicates a larger maximum span of the lesion. In our study, an increase in this value quantifies the expansion of the PCAT volume, which may originate from direct adipose tissue proliferation or from inflammation-induced edema and enlargement of adipose tissue and implies an increased activity of the PCAT, including an accumulation of inflammatory cells, such as macrophages, which release inflammatory factors that directly affect neighboring coronary arteries through paracrine effects, contributing to plaque formation and progression. Therefore, an increase in the value of original_shape_Maximum3DDiameter not only reflects the spatial expansion of PCAT but is also an indirect indicator of the local inflammatory state and altered biological activity associated with the progression of coronary artery disease. The GLRLM in the laplaciansharpening_glrlm_ShortRunLowGrayLevelEmphasis feature is a method for quantifying texture that examines the continuity of gray values in an image to analyze the texture characteristics and provides a wide range of information about the image texture by describing the length of time that the gray levels in the image appear in a certain direction. ShortRunLowGrayLevelEmphasis mainly reflects the texture details and gray-level distribution, emphasizing the short distance and low gray-level pixels in the image. The high value of ShortRunLowGrayLevelEmphasis indicates that there are a large number of continuous pixel sequences of low gray value and short length in the image, on the contrary, a low value of ShortRunLowGrayLevelEmphasis means that there are fewer darker textures in the image or that these textures occur in longer sequences. In coronary artery inflammation, the degree of infiltration and edema of the associated inflammatory cells alters the density and heterogeneity of the tissue, which in turn causes texture changes, and the ShortRunLowGrayLevelEmphasis value reflects such structural and textural changes, thus predicting plaque progression. The mean_glszm_GrayLevelNonUniformity feature measures the non-uniformity of the size distribution of consecutive groups of pixels with the same gray value in an image and can help to identify lesion status. The progression of coronary plaque leads to an increased local inflammatory response, which in turn affects the nature of the surrounding adipose tissue, and this change in the local environment can be captured in the values of this feature, reflecting changes in the structure and composition of the internal adipose tissue.

These features indicate that the radiomics model offered more nuanced information on fat heterogeneity and morphological variations in PCAT, thereby enhancing the prediction accuracy for RPP compared to the traditional plaque characteristics model. The utilization of this advanced radiomics approach is particularly advantageous in enhancing risk assessment. It facilitates the identification of patients at a higher risk of coronary plaque progression, which is pivotal for early and proactive medical intervention.

When combining PCAT radiomics features with clinical characteristics and plaque characteristics respectively, the AUC of the combined model increased only marginally and did not show statistical significance compared to the PCAT radiomics model. This suggested that the radiomics model had already captured the relevant data, proving to be sufficiently robust. Therefore, clinical characteristics and plaque characteristics had no additional contribution to the predictive value of RPP in this study.

The limitations of this study should be recognized. Firstly, this was a retrospective study with a relatively small sample size, which may hamper its reproducibility. Secondly, some patients received statins or other lipid-lowering therapy in the interval between CCTA, and the specific medication cycle and dose of each patient were different, which may have a certain impact on the results of the study. Thirdly, patients in our study who had revascularization prior to a follow-up CCTA were excluded, leading to selection bias among participants.

In conclusion, in the prediction of rapid plaque progression, the PCAT radiomics model outperformed the clinical model and plaque characteristics model. This offers a new perspective for early detection and intervention of rapid plaque progression.

## Data Availability

The datasets used or analyzed during the current study are available from the corresponding author upon reasonable request.

## Abbreviations

AUC: Area under the curve
CCTA: Coronary computed tomography angiography
DCA: Decision curve analysis
FAI: Fat attenuation index
GLRLM: Gray-level run-length matrix
GLSZM: Gray-level size-zone matrix
HU: Hounsfield units
LASSO: Least absolute shrinkage and selection operator
PB: Plaque burden
PCAT: Pericoronary adipose tissue
ROC: Receiver operating characteristic
ROI: Region of interest
RPP: Rapid plaque progression
SD: Standard deviations
